# Genome Wide Identification and Expression Profiling Indicate Expansion of Family I84 Protease Inhibitor *via* Gene Tandem Duplication and Divergence in Razor Clam *Sinonovacula constricta*

**DOI:** 10.3389/fimmu.2022.907274

**Published:** 2022-06-01

**Authors:** Sheng Liu, Youli Liu, Jiali Lu, Jinxia Mao, Zhihua Lin, Qinggang Xue

**Affiliations:** ^1^ Ninghai Institute of Mariculture Breeding and Seed Industry, Zhejiang Wanli University, Ningbo, China; ^2^ Zhejiang Key Laboratory of Aquatic Germplasm Resource, Zhejiang Wanli University, Ningbo, China; ^3^ Laboratory for Marine Fisheries Science and Food Production Processes, Pilot National Laboratory for Marine Science and Technology (Qingdao), Qingdao, China

**Keywords:** family I84 protease inhibitor, genome-wide identification, duplication, differentiation, host defense, mollusk

## Abstract

Family I84 protease inhibitors represent a novel family in the MEROPS peptidase database and are likely unique for molluscan host defense. Two Family I84 members, *scSI-1* and *scSI-2*, were reported from the razor clam *Sinonovacula constricta* in a previous research. In the present study, 12 additional genes, named *scSI-3* to *scSI-14*, were identified *via* genome wide sequence analyses. Among them, 10 genes were predicted to have a signal sequence, but one (*scSI-7*) was not. Besides, one sequence (*scSI-14*) was likely to encode a prematurely terminated peptide. The predicted mature peptides shared characteristics including 12 conserved cysteine residues, isoelectric points of 4.98 to 6.11, and molecular weights of 7.1 to 9.3 kDa with previously reported family members. Four motifs were characterized in 13 predicted mature peptides (with exception of scSI-14), which shared two to four conserved cysteine residues, are possibly to form two functional domain comprised 6 cysteine residues, respectively. At genomic level, all the 14 razor clam Family I84 genes were organized into 3 exons and 2 introns; 13 of them clustered in 3 regions of 100 kb on 3 separate chromosomes, suggesting tandem duplications of related genes. The promoter region of all the 14 genes was predicted to share some transcription factor binding sites, in particular those responsive to pathological and physiological stimuli, but no shared motifs were identified. Analyses also revealed differences in expression patterns among the genes. One gene in a tandem duplicated gene pairs usually showed a higher expression level than the other whereas non-tandem duplicated genes exhibited a higher degree of correlation in expression level. In addition, 8 of the 14 genes demonstrated higher level of expression in *Vibrio* tolerant clams than in non-tolerant clams following challenges with *Vibrio parahaemolyticus.* These results generated important information about the evolution of Family I84 protease inhibitors in *S. constricta*.

## Introduction

Protease inhibitors are a special group of molecules ubiquitously present in all organisms and one of their functions is believed to play a role in host immunity (1, 2). The involvement of protease inhibitors in molluscan host defense are indicated by the findings of homologous genes of several known protease inhibitors, such as the tissue inhibitor of metalloprotease and Kazal-type serine protease inhibitor in the Pacific oyster *Crassostrea gigas* (3), the bay scallop *Argopecten irradians* (4), and the Manila clam *Ruditapes philippinarum* (5). More direct evidence for protease inhibitors’ functioning in the immunity of mollusk species, however, comes from a series of research on the Family I84 protease inhibitors in the MEROPS peptidase and peptidase inhibitor classification system (http://www.ebi.ac.uk/merops).

The Family I84 in MEROPS was first created to host cvSI-1 and cvSI-2 from the eastern oyster *Crassostrea virginica*, the first protease inhibitors to be purified and characterized in a molluscan species (6–8). More family members have been identified later at the gene sequence level in several other mollusks including oysters (*C. gigas*), mussels (*Mytilus galloprovincialis*), clams (*Sinonovacula constricta* and *Meretrix meretrix*), and abalones (*Haliotis discus hannai*) (9–11). A more recent research indicates that Family I84 is likely a multigenic family present exclusively in the Phylum Mollusca and the family has expanded significantly in certain molluscan lineages (11, 12).

Family I84 genes encode peptides that have a signal peptide at the N-terminus. Mature molecules have a molecular weight of about 8 kDa and consist of around 70 amino acid residues including 12 conserved cysteines that are speculated to form 6 intramolecular disulfide bonds (7, 10, 11, 13). They selectively inhibit the enzyme activity of serine type of proteases in particular that in the subtilisin family (6, 7). Several features of Family I84 protease inhibitors indicate their role in mollusk immunity. Purified cvSI-1, for example, inactivates the virulence protease of *Perkinsus marinus*, the protozoan parasite of eastern oysters and inhibits the parasite’s propagation *in vitro* (6, 7). At the same time, levels of plasma subtilisin inhibitory activity and related gene expression are significantly higher in oyster populations selected for increased resistance or oyster species inheritably resistant to *P. marinus* than in oysters highly sensitive to the parasite (13). In addition, the expression of Family I84 genes in oysters, clams, and mussels are detected to be up-regulated by injection of bacterial and protozoan pathogens and pathogen associated molecular patterns (PAMPs) (9, 11, 14, 15).

The razor clam *S. constricta* is a representative species of bivalve mollusks inhabiting on the muddy intertidal zone. Two Family I84 genes, *scSI-1* and *scSI-2*, are identified from the clam through cDNA cloning and sequencing (9). The encoded peptides are predicted to share critical molecular characteristics of the Family I84 protease inhibitors. The clam plasma is also detected to contain the signature protease inhibitory activity of the Family I84 protease inhibitors. Besides, the expressions of the 2 genes are upregulated the clams following challenges by *Vibrio harveyi* and extreme physiochemical factors, suggesting their function in host defense. In addition to *scSI-1* and *scSI-2*, searches into transcriptomic sequence databased have also revealed possibilities of the presence of more Family I84 members in the genome of *S. constricta*. However, the entire repertoire of the family in the razor clam remained unknown.

The availability of the genome sequencing data for *S. constricta* has made it possible to study a gene family *via* genome wide identification (16). This kind of genomic studies in combination with gene expression profiling at the transcriptomic level can generate important information about functions and evolution of the gene family of interest (17, 18). Thus, we aimed in the present research at: (1) determining total number, structure and chromosome distribution of the Family I84 genes in the *S. constricta* genome; (2) analyzing the phylogenetic relationship between the identified *S. constricta* genes with known Family I84 members; (3) decoding the mechanisms of molecular evolution of Family I84 genes in *S. constricta*; and (4) profiling expressions of Family I84 genes in *S. constricta* at different developmental stages and following bacteria challenges. Results of the research will provide important information about the molecular and functional evolution of Family I84 protease inhibitors in mollusks.

## Materials and Methods

### Family I84 Gene Search and Analysis

BLAST search into the *S. constricta* genome assembly at the GenBank (accession number GCA_009762815.1) was performed in May 2021 using the tBLASTn program with the 12 Family I84 members from 5 mollusk species (cvSI-1, cvSI-2 and cvSI-3 from *C. virginica*; scSI-1 and scSI-2 from *S. constricta*; mgSI-1, mgSI-2 and mgSI-3 from *M. galloprovincialis*; mmSI-1 and mmSI-2 from *M. meretrix*; hdhSI-1 and hdhSI-2 from *Haliotis discus hannai*) as queries (9, 11, 12). Sequence hits with E<0.01 were downloaded and checked for the presence of a complete open reading frame (from the initial ATG to the stop codon). Partial sequences were complemented to their full length by comparison with transcriptome assembly (16).

The retrieved sequences were verified by PCR amplification of the predicted open reading frame (ORF). The PCR amplifications were done using cDNA synthesized from the digestive gland RNAs as templates and primers listed in **Supplementary Table 1**. For sequences that the PCR using cDNA failed to generate an expected amplicon, the PCR verification was carried out using the genomic DNA from the gill tissues as the templates. Tissue samples for RNA and genomic DNA preparations were collected from healthy adult clams maintained in seawater at 20°C and salinity at 20 psu. Total RNAs and genomic DNA were extracted from related tissues respectively using an RNA isolation kit (Omega, USA) and a DNA extraction kit for marine animals (Tiangen, Beijing, China) according to the manufacturer’s instructions. DNA and RNA concentrations were determined using a micro-spectrophotometer (Nanodrop, Allsheng) and the integrity was checked by electrophoresis in 1.2% agarose gel. The cDNA templates were synthesized using the Prime Script™ RT kit (Takara, Japan).

ORFs were predicted with the ORF Finder at NCBI (https://www.ncbi.nlm.nih.gov/orffinder/). Multiple sequence alignments and sequence percent identity calculations were done using Clustal Omega (https://www.ebi.ac.uk/Tools/msa/clustalo/). Aligned sequences were visualized using Jalview v2.11.1.0 (19). Signal sequences were predicted using SignalP 5.0 (www.cbs.dtu.dk/services/SignalP/) and SecretomeP 2.0. Molecular weight (MW) and theoretical isoelectric point (pI) were calculated using Protparam at ExPASy (https://web.expasy.org/protparam/).

### Phylogenetic Tree Construction

To determine the evolutionary origin of razor clam Family I84 genes, mature peptides sequence of all Family I84 members in razor clam and 15 other identified members in oysters, mussel, clams and abalone were used to construct a phylogenetic tree. Multiple sequence alignment were conducted with Muscle method (20); the best evolutionary model were predicted and model WAG + Gamma + Invariant showed the best parameters and then maximum likelihood (ML) phylogenetic tree was constructed using MEGA6 with a bootstrap of 1,000 replicates (21).

### Motif, Gene Structure, Chromosomal Distribution and Duplication Analysis

The *de novo* motif identification was carried out in mature peptides using the MEME suite (https://meme-suite.org/meme/tools/meme) with the default parameter. Gene structure including length and number of exons and introns were determined by BLAST the full length of the candidate genes on the genome assembly. General feature format (gff) files containing these genes’ annotation information were then generated. Gene structure and motif patterns were visualized *via* Toolbox for Biologists (TBtools) v1.05 (22). Chromosomal distributions of the Family I84 genes were visualized using the TBtools.

Gene duplication was determined based on two criteria: >70% coverage of the longer sequence and >70% similarity in a pairwise alignment of 2 sequences without signal peptides (23, 24). Duplicated genes separated by five or fewer genes in a 100-kb chromosomal fragment were considered tandem duplicated genes (24).

### Positive Selection and Site Conservation Detection

Simple Ka/Ks Calculator in TBtools were used to calculate the number of substitutions per synonymous site (K_S_) and nonsynonymous site (K_A_) for duplicated gene pairs (22). CodeML method in EasyCodeml v1.4 was used to test whether the single sites were under positive selection (25). Briefly, nucleotide sequences of Family I84 genes in razor clam were aligned with codon-based method in MEGA, seven codon substitution models encompassing M0 (one-ratio), M1a (nearly neutral), M2a (positive selection), M3 (discrete), M7 (beta), M8 (beta and ω >1) and M8a (beta and ω = 1) were tested. Four likelihood ratios (M0 vs M3, M1a vs M2a, M7 vs M8 and M8a vs M8) were performed and compared using a likelihood ratio test (LRT).

The codon usage frequencies of TGT or TGC for cysteine were calculated on the CDS of all protein coding genes on the genome (GenBank assembly accession: GCA_009762815.1) and the Family I84 genes in *S. constricta* using the “cusp” tool in the mEMBOSS package (26). The codon usage bias for the twelve conserved cysteine were carried out by Chi-square test assuming a binomial distribution.

### Promoter Sequence Analysis

The 2kb sequence upstream of the translation initiation site was extracted as a candidate promoter region according to the Family I84 gene structure and gff file. Multiple sequence alignment for promoter sequences were conducted with Clustal Omega program (https://www.ebi.ac.uk/Tools/msa/clustalo/) and identities among sequences were calculated and compared. The sequence were then analyzed using simple MEME Wrapper in TBtools v1.05 with default parameters for motif identifications (22). Potential transcription factors and their binding sites were predicted using Alibaba 2.1 program in the GeneXplain online tool with the pairSIM being set at 64 and the min matrix conservation at 80% (http://gene-regulation.com/pub/programs/alibaba2/index.html).

### Transcriptome Assembly and Gene Expression Profiling

Transcriptomic sequence data of 66 samples covering *S. constricta* at developmental stages, under variable challenges and populations differed in resistance to *V. parahaemolyticus* were retrieved from the NCBI database (**Supplementary Table 2**). The developmental stages included egg, four cells, blastulae, gastrulae, trochophore, D-shaped larvae, umbo larvae and juvenile. Environmental stressors for the clam challenges included ammonia nitrogen and temperature. Sequence data for clam populations different in *V. parahaemolyticus* resistance were generated (27). The retrieved RNA-seq reads were mapped to the *S. constricta* genome using Hisat (V2.1.0) (28), and assembled using StringTie (29). Gene expressions were normalized and reported in Transcripts Per Kilobase of exon model per Million mapped reads (TPM) (30). The correlation in expression levels between Family I84 genes was calculated in the transcriptomic sequence data of *V. parahaemolyticus* resistant and sensitive clams, 15 individuals each, after the bacterial injection using Pearson analysis with the corrplot package in RStudio software.

## Results

### Family I84 Gene Repertoire in the Clam Genome

**Figure 1:**
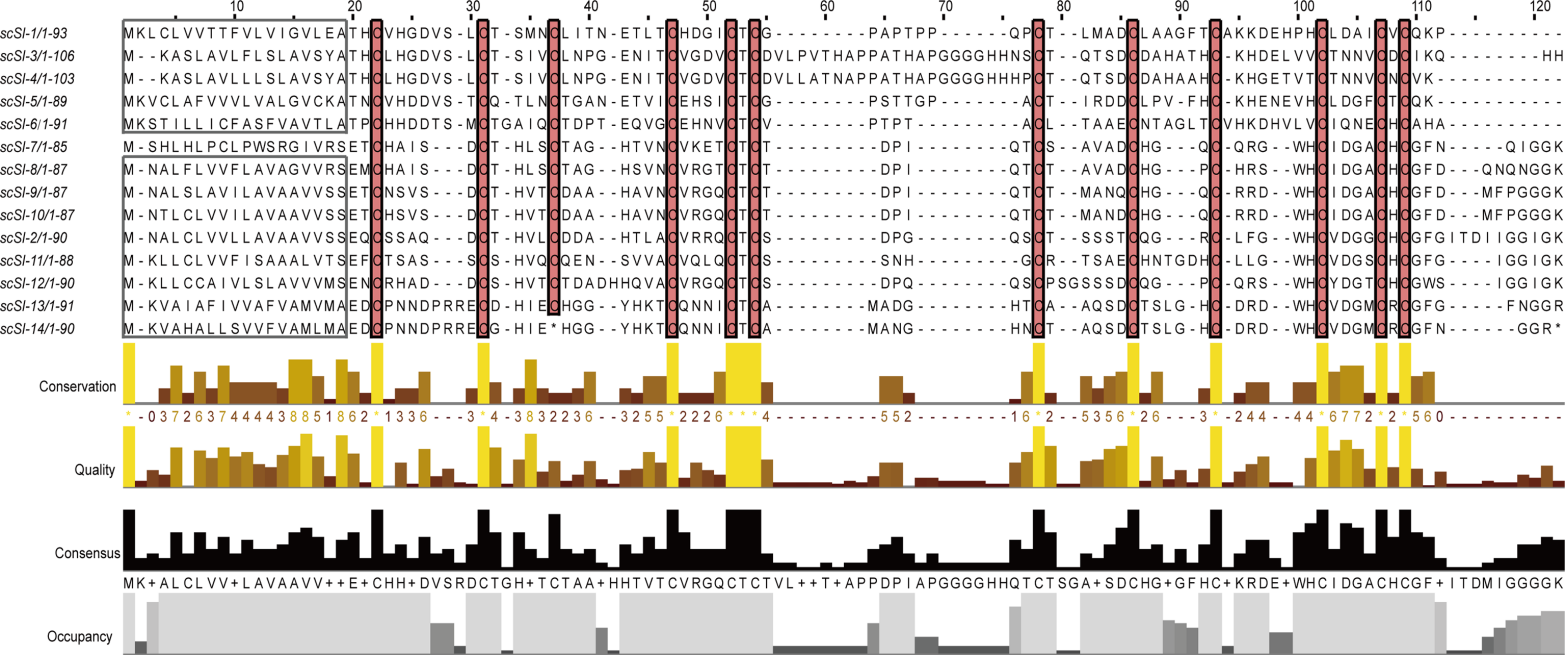
Peptide sequence alignments of the fourteen Family I84 genes from razor clam, two formally characterized members *scSI-1* and *scSI-2* were included. Predicted signal peptide are framed, conserved cysteine residues are highlighted. Conservation and consensus were shown of the residues. * means the stop codon of peptide.

Twelve new Family I84 protease inhibitor genes in addition to *scSI-1* and *scSI-2* were identified in the *S. constricta* genome and named *scSI-3* to *scSI-14*, making the razor clam Family I84 gene repertoire being composed of 14 genes. The 12 newly identified genes encompassed an ORF ranging from 258 to 321 bp that encoded peptides from 85 to 106 amino acids. *ScSI-14* encoded a much shorter peptide (34 amino acids) than the other members due to a mutation from TGT to TGA leading to premature termination (**Table 1**; **Supplementary Figure 1**). All genes but *scSI-7* were predicted to have a signal peptide of 17 to 19 amino acids and a mature peptide length ranging from 69 to 89 amino acids. The 12 conserved cysteine were identified in all mature peptides, but the array was highly variable as **C**X_5-8_ **C**X_4-5_ **C**X_7-9_ **C**X_4_ **C**T**C**X_6-23_ **C**X_5-7_ **C**X_3-6_ **C**X_5-8_ **C**X_4_ **C**X**C** (**Figure 1**). Mature peptides had a predicted PI of 4.98~6.11 and molecular weight of 7.1~ 9.3 kDa. The highest and lowest identities of these members with Family I84 type molecule (cvSI2) were 46.77% and 20.31%, respectively (**Table 1**), and the sequence identity among the 14 razor clam members ranged from 24.14% to 87.21% (**Supplementary Table 3**). All 14 genes excluding *scSI-7* and *scSI-14* of the razor clam Family I84 repertoire were amplified in PCR with cDNA templates. A 1658 bp and a 2596 bp fragments encompassing the predicted ORF of *scSI-7* and *scSI-14* were amplified in PCR reactions with genomic DNA templates.

**Table 1:** Sequence parameter of full repertoire of razor clam Family I84 protease inhibitor.

Gene	CDS	AA	Signal peptide	Mature AA	Cys	PI	MW(kD)	Identity		
								cvSI-1	cvSI-2	cvSI-3
scSI-1	282	93	19	74	12	5.52	7.8	32.35	30.77	27.27
scSI-3	321	106	17	89	12	5.76	9.3	25.37	20.31	21.54
scSI-4	312	103	17	86	12	5.89	8.8	21.21	20.63	20.31
scSI-5	270	89	19	70	12	4.98	7.6	32.31	25.81	23.81
scSI-6	279	92	19	73	12	5.17	7.6	25.37	24.62	23.08
scSI-2	273	90	18	72	12	5.62	7.5	30	45.16	37.1
scSI-7*	258	85	—	67	12*	6	7.1	28.33	43.55	35.48
scSI-8	264	87	18	69	12	6.11	7.3	31.67	43.55	38.71
scSI-9	264	87	18	69	12	5.85	7.4	30	45.16	38.71
scSI-10	264	87	18	69	12	5.74	7.4	30	46.77	40.32
scSI-11	267	88	18	70	12	6	7.3	27.42	30.65	32.26
scSI-12	273	90	18	72	12	5.74	7.7	25.81	40.32	38.71
scSI-13	276	91	18	73	12	5.51	8	36.36	33.87	30.16
scSI-14**	270	34	18	16	—	—	—	—	—	—

*The 18 AA of N terminal of this peptide were removed for latter comparison.

**There is a mutation from TGT to TGA leading to the premature termination of this gene.

### Phylogenetic Tree

**Figure 2:**
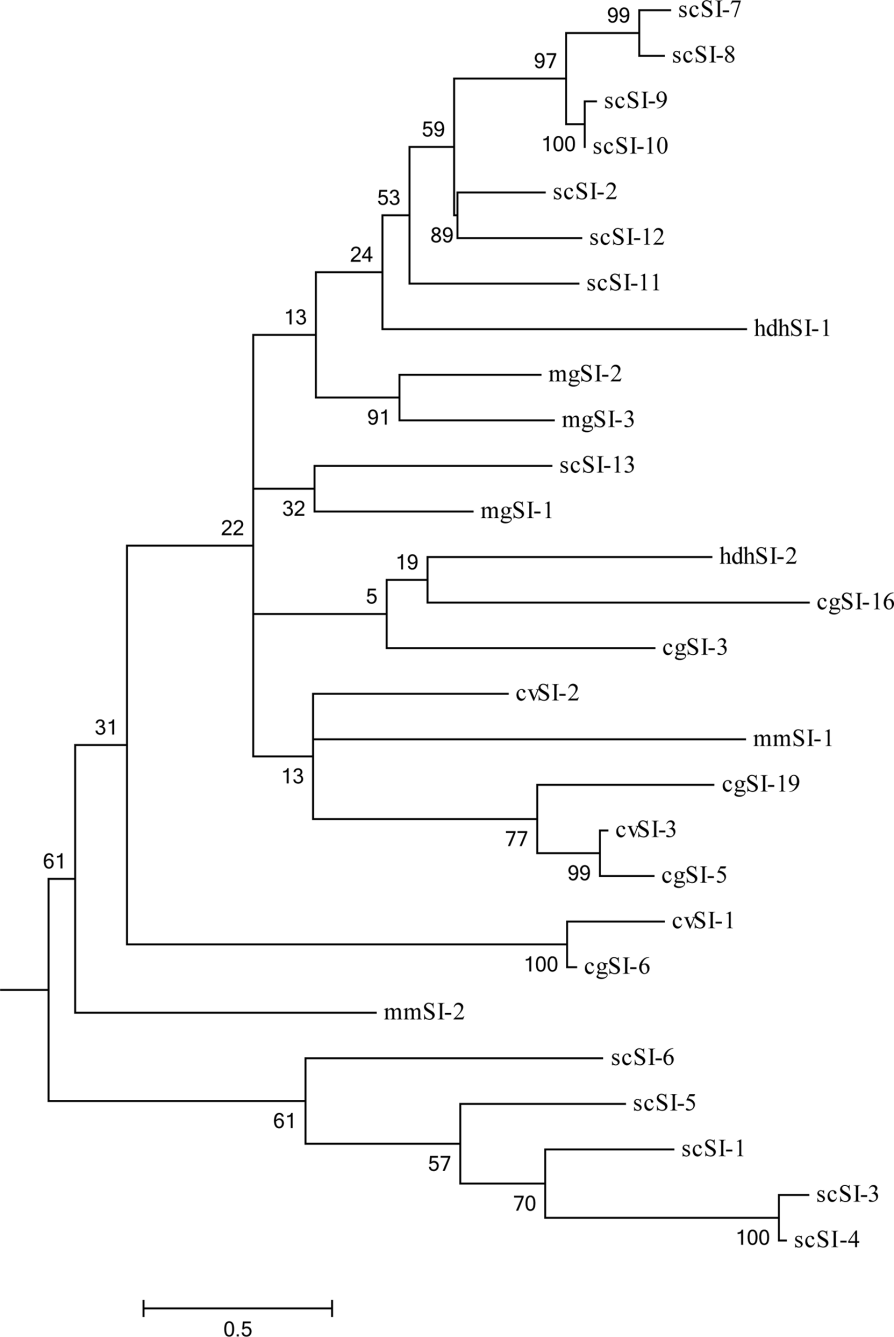
Maximum Likelihood (ML) phylogenetic tree containing 28 members of Family I84 protein sequences without signal peptide. The accession numbers were AAZ41364.1, BAH20735.1 and AQM52281.1 for cvSI-1, cvSI-2 and cvSI-3 in *Crassostrea virginica*; UBF40522.1, UBF40523.1, UBF40524.1 for mgSI-1, mgSI-2, and mgSI-3 in mussel *Mytilus galloprovincialis*; ATY73923.1, ATY73924.1 for scSI-1, scSI-2 in clam *Sinonovacula constricta*; UBF40520.1, UBF40521.1 for mmSI-1, mmSI-2 in clam *Meretrix meretrix*; QPM65700.1, QPM65701.1 for hdhSI-1, hdhSI-2 of abalone, *Haliotis discus hannai* used in this phylogeny analysis, the other 11 sequences from clam *S.constricta* were acquired in the present study. Numbers beside the internal branches indicate bootstrap values based on 1000 replications, the 0.5 scale indicates the genetic distance.

An ML phylogenetic tree containing 28 members’ mature peptide of Family I84 protein sequences was constructed (**Figure 2**). These sequences were grouped into two major nodes including 23 and 5 members, respectively; however, the bootstrap values poorly supported the two nodes all the time. Sequences from the same or closely related species tended to cluster together and the first node included one cluster which comprised scSI-1, scSI-3, scSI-4, scSI-5 and scSI-6. The other node contained four clusters and they included 7 genes (scSI-2, scSI-7, scSI-8, scSI-9, scSI-10, scSI-11 and scSI-12), 3 genes (cvSI-3, cgSI-5 and cgSI-19), 2 genes (mgSI-2 and mgSI-3), and 2 genes (cvSI-1 and cgSI-6), respectively.

### Motifs and Gene Structure

**Figure 3:**
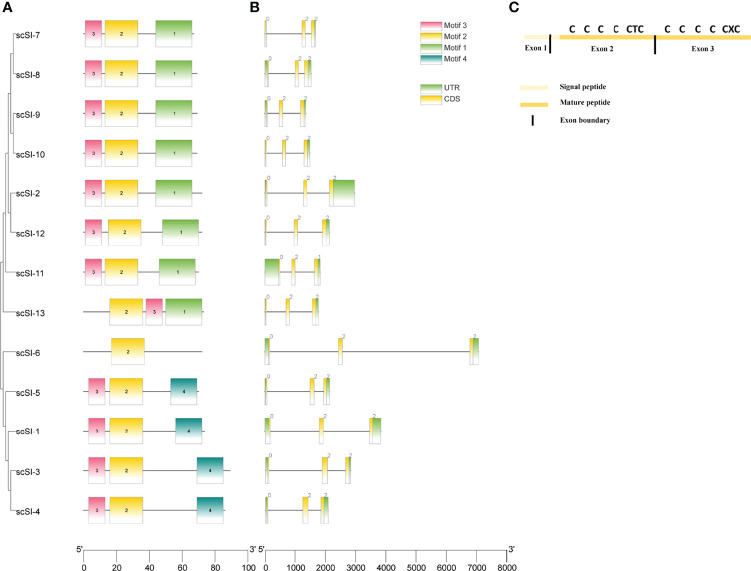
Mature polypeptide motifs and gene structure for all Family I84 genes excluding the scSI-14. **(A)** Polypeptide motifs of these gene members, different color blocks are motifs and the 1,2,3,4 in the block represent the number of motif. Motif 1: QCQRGWHCIDGACHCGFDINGGG, Motif 2: CDAGHTVTCVRGQCTCTDPIQ, Motif 3: TCGDVSDCTHV, Motif 4: KHDEEVHCLDN VCDCQK; **(B)** Gene structure of all the I84 family genes and 0, 1, 2 in the block are the phase for translation; **(C)** Cysteine pattern and their distribution on exons for all of the members.

A total of 4 motifs were characterized in 13 mature peptides (**Figure 3A**). Among them, motif 1 and motif 2 each covered four conserved cysteine residues displaying in arrays of C-X_5_-C-X_4_-C-H-C and C-X_7_-C-X_4_-C-T-C respectively. Motif 3 and motif 4, however, covered different numbers of conserved cysteines with the former containing 2 and the latter 3 in an array of C-X_5_-C and C-X_4_-C-D-C, respectively. Most sequences comprised three motifs, of which seven genes (scSI-2, scSI-7, scSI-8, scSI-9, scSI-10, scSI-11, scSI-12 and scSI-13) contained motif 1, 2 and 3; four genes (scSI-1, scSI-3, scSI-4 and scSI-5) contained motif 2, 3 and 4, but scSI-6 contained only one (motif 2), which were shared by all razor clam Family I84 members.

All genes consisted of three exons and two introns, phase of exons for gene translation were 0, 2 and 2, with exception for *scSI-11* (0, 2 and 1); their gene length spanned from 1485 bp (*scSI-10*) to 7077 bp (*scSI-6*) on the genome (**Figure 3B**). The 3 exons coded for different parts of the whole peptide, with first exon for part of the signal peptide, the second exon for the remaining part of signal peptide and the N-terminal segment coving the first to sixth conserved cysteine, and the third exon for the C-terminal segment covering the 6 remaining conserved cysteine residues (**Figure 3C**).

### Gene Chromosomal Location and Tandem Duplication

**Figure 4:**
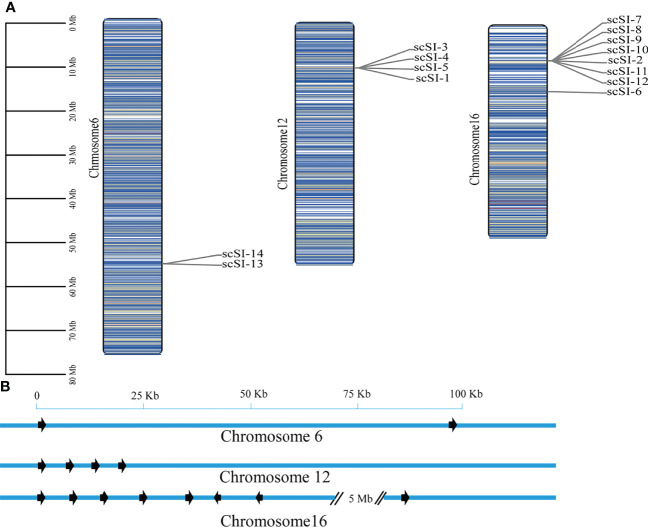
Chromosome distribution of all the Family I84 genes in razor clam, **(A)** Global view of these genes on three chromosomes, the darker of the bar, the higher density for genes around it; **(B)** Regional appearance of gene clusters on chromosomes, the arrow are transcriptional direction of genes.

The 14 Family I84 genes of *S. constricta* were mapped on three chromosomes- chromosome 6, chromosome 12 and chromosome 16 (**Figure 4**). For the 8 genes in chromosome 16, 7 (*scSI-2*, *scSI-7*, *scSI-8*, *scSI-9, scSI-10, scSI-11* and *scSI-12*) were localized in a 52 kb genome region, and the other (*scSI-6*) was about 5M bp away from the 7 gene cluster. On the other hand, four genes (*scSI-1* and *scSI-3*, *scSI-4*, and *scSI-5*) clustered in a 23 kb genome region on chromosome 12, and 2 genes (*scSI-13* and *scSI-14*) in a 95 kb region on chromosome 6.

Gene duplications were detected for the razor clam Family I84 genes and based on the defined criteria, 8 genes (57.1%) in three clusters were determined to be tandem duplicated (**Table 2**). Three tandem duplicated gene clusters included scSI-3 and scSI-4; scSI-7, scSI-8, scSI-9 and scSI-10; scSI-14 and scSI-13, respectively.

**Table 2:** Coverage and similarity among peptide sequences of Family I84 genes.

	scSI-3	scSI-4	scSI-7	scSI-8	scSI-9	scSI-10	scSI-13
scSI-3	100%						
scSI-4	97%/88%	100%					
scSI-7	/	/	100%				
scSI-8	/	/	97%/88%	100%			
scSI-9	/	/	97%/79%	100%/78%	100%		
scSI-10	/	/	97%/81%	100%/79%	100%/98%	100%	
scSI-13	/	/	/	/	/	/	100%
scSI-14*	/	/	/	/	/	/	98%/89%

*Coverage and similarity between scSI-13 and scSI-14 are based on CDS sequence due to the premature termination of scSI-14.

### Positive Selection and Site Conservation

For all the duplicated gene pairs, Ka/Ks for the coding sequence (excluding signal peptide) ranged from 0.16 (*scSI-8* with *scSI-10*) to 0.57 (*scSI-3* with *scSI-4*) (**Table 3**). Site model analyses did not detect positive selection sites in the razor clam Family I84 genes, however, LRT P-value between M0 and M3 models was lower than 0.000 (**Table 4**).

**Table 3:** Ka/Ks of duplicated genes.

Seq_1	Seq_2	Ka	Ks	Ka/Ks
scSI-3	scSI-4	0.07	0.12	0.57
scSI-7	scSI-8	0.06	0.24	0.25
scSI-7	scSI-9	0.21	0.74	0.28
scSI-7	scSI-10	0.18	0.88	0.21
scSI-8	scSI-9	0.23	1.29	0.18
scSI-8	scSI-10	0.2	1.29	0.16
scSI-9	scSI-10	0.02	0.1	0.19

**Table 4:** Positive selection analyses of razor clam Family I84 genes based on site model.

Model	ln*L*	Null	LRT	df	*P*-value	Positive selected sites
M0	-1903.788	NA				
M3	-1804.468	M0	198.638	4	0.000	[]
M1a	-1845.595	NA				
M2a	-1845.595	M1a	0.0	2	1.000	[]
M7	-1810.314	NA				
M8	-1810.314	M7	1.559	2	0.999	[]
M8a	-1810.314	M8	0.0	1	1.000	[]

NA means no Null hypothesis were compared with corresponding model, [] means no Positive selected sites were detected.

**Figure 5:**
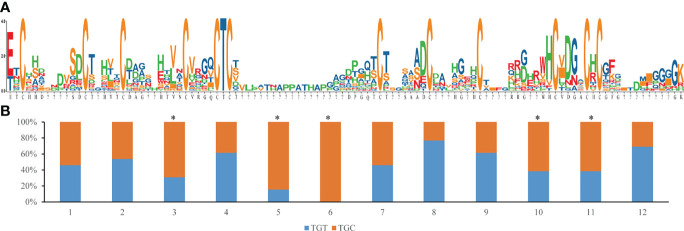
Mature peptide sequence variability and conservation of cysteine residues, **(A)** Sequence logo of 13 mature peptide, the larger of the letter, the higher proportion of the amino acid in the position,”?” means the position are not shared for all of the sequence; **(B)** Codon usage percentage of TGT or TGC for 12 conserved cysteine residues, the asterisk means that the ratio in that residue are deviated from the average of the whole genome codon usage frequency (0.646 for TGT and 0.354 for TGC).

The codon usage frequency of cysteine was 0.646 for TGT and 0.354 for TGC in all protein coding genes from the *S. constricta* genome. Five of the twelve conserved cysteines were significantly deviated from the average (**Figure 5**). Specially, for the fifth and sixth cysteine, codon preference was TGC, forming the most conserved region (C-T-C) of Family I84 genes.

### Promoter Sequence Feature

The highest and lowest identities among 14 Family I84 members’ promoter sequences were 51.73% and 29.32%, respectively (**Supplementary Table 4**). No motifs were shared among all razor clam Family I84 members; however one motif was shared among *scSI-3* with *scSI-4* and another motif was shared by *scSI-2*, *scSI-5*, *scSI-6*, *scSI-7*, *scSI-8*, *scSI-9* and *scSI-10* (**Supplementary Figure 2**). In total, 14 Family I84 members had binding sites for 14~29 transcription factors, which may functioned in response to physiological and pathological stimuli (**Supplementary Table 5**). Briefly, 13 razor clam Family I84 genes (92.9%) had one to seven Oct (Octamer-binding protein) binding sites; 7 razor clam Family I84 genes (50%) had one or more IRF (Interferon regulatory factor) binding sites. C/EBP (CCAAT/enhancer binding protein) and HNF (Hepatocyte nuclear factor) were found in 14 and 13 family I84 genes, respectively. Two to five CREB (Cyclic AMP-responsive element-binding protein 1) binding sites existed in 5 Family I84 genes. Altogether, the transcription factors binding sites analysis indicated that razor clam Family I84 genes could involve in different immune and inflammatory responses.

### Gene Expression Patterns

**Figure 6:**
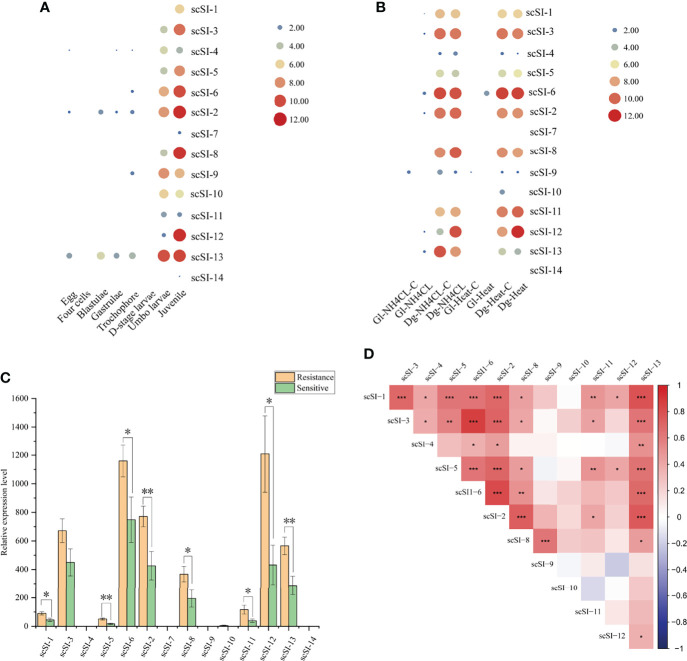
Gene expression patterns and co-expression of Family I84 genes. **(A)** Expression patterns of genes at different development stages of egg, four cells, blastulae, gastrulae, trochophore, D-stage larvae, umbo larvae and juvenile after log_2_ transformation; **(B)** Transcription level of Family I84 genes under the stress of ammonia nitrogen (180 mg/L with 0 mg/L as control) in gill (Gl-NH_4_CL, Gl-NH4CL-C) and digestive gland (Dg-NH_4_CL, Dg-NH4CL-C); high temperature (32°C with 22°C as control) in gill (Gl-heat, Gl-heat-C) and digestive gland (Dg-heat, Dg-heat-C) after log2 transformation; **(C)** Gene expression level for resistant and sensitive groups (n = 15 per group) of razor clam juveniles after *V. parahaemolyticus* infection, one and two asterisk represent the significant level lower than 0.05 and 0.01, respectively; **(D)** Co-expression analysis of I84 family genes in resistant and sensitive groups (n = 30 in total) of juveniles under *V. parahaemolyticus* infection, the darker of the block, the much stronger correlation are between them, *, ** and *** means the p valve for correlation coefficient lower than 0.05, 0.01 and 0.001, respectively.

The Family I84 genes were not detected in the developmental stages before D-shaped larvae except for *scSI-2*, *scSI-4*, *scSI-*6, *scSI-9* and *scSI-13*. However, all genes except for *scSI-7* and *scSI-14* were expressed in the umbo larvae and juveniles, and the expression levels increased gradually (**Figure 6A**).

Under the stress of ammonia nitrogen (180 mg/L with 0 mg/L as control) and high temperature (32°C with 22°C as control), Family I84 genes were hardly expressed in gills, but were much higher in the digestive gland under control or experiment group. *ScSI-1*, *scSI-2*, *scSI-3*, *scSI-6 scSI-8*, *scSI-11* and *scSI-12* showed higher expression levels than *scSI-4*, *scSI-7*, *scSI-9*, *scSI-10* and *scSI-14*, but there was no significant difference between the stress group and the control group (**Figure 6B**).

For resistant and sensitive groups of razor clam juveniles after *V. parahaemolyticus* infection, expression levels of *scSI-4*, *scSI-7*, *scSI-9*, *scSI-10* and *scSI-14* were much lower than the other nine Family I84 members (**Figure 6C**). Significantly higher gene expression was observed in the resistant group than in the sensitive group for *scSI-1*, *scSI-5*, *scSI-6*, *scSI-2*, *scSI-8*, *scSI-11*, *scSI-12* and *scSI-13*.

Correlation coefficient of gene expression of tandem duplicated Family I84 gene pairs (*scSI-3* with *scSI-4, scSI-8* with *scSI-9, scSI-9* with *scSI-10*) were lower than 0.4 and not significantly different, while correlation coefficient between *scSI-8* with *scSI-9* were 0.63 (p<0.001). For non-tandem duplicated genes, a high correlation was observed between *scSI-3* and *scSI-6* (r=0.87, p<0.001), *scSI-2* and *scSI-6* (r=0.80, p<0.001), *scSI-1* and *scSI-13* (r=0.77, p<0.001), *scSI-2* and *scSI-13* (r=0.74, p<0.001) and *scSI-2* with *scSI-8* (r=0.71, p<0.001), respectively (**Figure 6D**).

## Discussion

The Family I84 in the MEROPS peptidase database consists of novel and mollusk-specific serine protease inhibitors (6, 7, 13). Two Family I84 genes, *scSI-1* and *scSI-2*, have been cloned and their expressions are upregulated in *S. constricta* following challenges with *Vibrio harveyi* (9). A better understanding of the Family I84 in the razor clam is now achieved after the identification of 12 additional genes in the present study. The analyses of the entire Family I84 gene repertoire at the genome and transcriptomic levels have generated important information about the function and evolution of Family I84 protease inhibitors.

### Family I84 Protease Inhibitors Are Diverse Yet Conserved in Certain Characteristics

The identification of the *S. constricta* Family I84 gene repertoire reveals the extreme variability of the family members in overall amino acid sequence. The 14-razor clam Family I84 genes encode the peptides that share a sequence identity between each other varying from 24.14% to 87.21%, reflecting the molecular diversity of the Family I84 protease inhibitors within a same species. At the same time, the razor clam Family I84 members also show great degree of difference in amino acid sequences with their homologs in other species (e.g., 20.3%-46.8% identities with the family type member cvSI-2), indicating the molecular variability between the family orthologs in different species. Although molecular diversity seems to be a defining feature in protease inhibitors, the degree of diverseness of the Family I84 is still greater than most other protease inhibitors families including those from molluscan species (e.g., the kazal-type protease inhibitors from clam and scallop (4, 5, 31). In addition, the razor clam Family I84 appears to consist of 2 nonfunctionalized genes, *scSI-7* that lacks signal sequence and *scSI-14* that encodes a premature peptide, further increasing the family’s diversity. The extremity of Family I84 protease inhibitors in molecular diversity likely reflects the active evolution, which in turn suggests the functional significance, of the mollusk-specific protein family. It should be noted that the diverseness of Family I84 molecules as indicated by the range of sequence identity between intraspecific family members seems to be greater in *S. constricta* than in several other species such as *C. virginica*, *C. gigas*, and *M. galloprovincialis* (12). This difference in identity among intraspecific family sequences between the razor clam and other species may stem at the lack of recognition of the entire gene repertoire in the latter. Genome wide identification of the Family I84 genes in more species should provide valuable information.

While the Family I84 protease inhibitors are greatly diverse in the overall amino acid sequences, they are conserved in gene structure and cysteine residues. Similar to the cvSI-1 gene in eastern oyster, all the razor clam Family I84 genes consist of three exons and two type 1 introns (32). In addition, most mature peptides contained 12 conserved cysteines with a relatively conserved arrangement in the sequence. Intriguingly, the 12 cysteines are constantly encoded in the second and third exons with 6 in each, suggesting 2 functional modules being encoded by 2 separate exons. However, unlike the Kazal type protease inhibitors where 2-12 repeated modules with a similar 6-cysteines array form a “multiheaded” protease inhibitor (33), the 2 possible modules in Family I84 protease inhibitors differ from each other in the cysteine arrangement patterns and thus likely differ functionally as well (5, 31, 34). It is speculated that the 12 conserved cysteine residues in the Family I84 members form intramolecular disulfide bridges (Xue et al, 2009). Therefore, the conservation of cysteines in the family members appears to be necessary for the proteins to maintain a functional conformation.

### Family I84 Genes Expanded *via* Tandem Duplication

Gene distribution in the genome provide important information about the evolution of Family I84 genes. It is notable that 13 of the 14 razor clam Family I84 genes clustered in 3 regions of 100 kb on 3 separate chromosomes, and 8 genes meet the criteria of tandem duplication. The phenomenon that multiple genes present closely in the genome is also reported in the Pacific oyster Family I84 genes (12). Therefore, Family I84 may have undergone expansion *via* tandem duplication after speciation of bivalves. Interestingly, tandem duplications have been observed in expansion of several host immunity related gene families including antimicrobial peptides and toll-like receptors (17, 35, 36). Unlike the cysteine-rich antimicrobial polypeptide-big defensins where the species associated monophyletic topology on a phylogenetic tree indicates an exclusive species-specific expansion *via* tandem duplication; however, we find in the present research that the Family I84 genes are not always grouped in accordance with the species. A similar phenomenon has been reported in an EST contig based research (12). Some genes of the Family I84 may thus have duplicated before the speciation of bivalves and the speculation appears to conform to the distribution of the razor clam genes in 3 different chromosomes.

### Positive Selection Was Not Detected in Duplicated Genes

Positive selection is believed to play an important role in driving the diversification of the duplicated genes during the evolution of some protease inhibitors (31, 37, 38). Yu etal. (39) reported a Ka/Ks ratio of 1.4 in the eastern oyster cvSI-1 gene. We calculated a Ka/Ks ratio of 1.3 for *scSI-1* in a razor clam population with 150 individuals (data not shown). These research results seem to be indicative for the involvement of positive selection in the evolution of at least some Family I84 genes. However, no positively selected sites were detected in razor clam Family I84 genes in the present research; the significantly lower than 0.001 p-value between M0 and M3 models could only illustrate that selection pressure of different sites are not constant (25). The reason for failing to observe positive selection of Family I84 genes from razor clam, are possibly that these genes have been duplicated several times, as shown by the sequence identity and Ka/Ks for duplicated gene pairs, thus, diversifying time may be insufficient to accumulate enough nonsynonymous mutations in these paralogs (40). However, it cannot be concluded that the evolution of the Family I84 gene does not involve positive selection, on the contrary, the possibility of the gene family evolving through positive selection still exists and identification of more Family I84 orthologs in various mollusk species are required in future studies.

### Gene Expression Variations Suggested Functional Divergence

It is noteworthy that expression of Family I84 genes, especially duplicated genes, diversified significantly. Generally, only one gene has a high expression level within tandem duplicated gene pairs, and gene expression between non-tandem duplicated genes has a high correlation. The difference between these genes could reach up to 100-fold when we excluded the above mentioned low expression genes. It had also been observed in eastern oyster, where *cvSI-1* and *cvSI-2* expression levels are nearly 1000 times that of *cvSI-3* (12). *ScSI-7* and *scSI-14* were barely expressed in the analyzed transcriptome, we speculate that loss of the signal peptide of scSI-7 and premature termination of *scSI-14* peptide, may drive their loss of function and silencing compared with their tandem duplicated partner. That is also detected in immune-related multi-gene families, some duplicated genes may have undergo frame-shift or pseudogenization during evolution (17, 36). Similarly, a number of TLR genes expanded in the Pacific oyster genome, but expression patterns of tandem-linked TLR genes in the same scaffold varied dramatically during oyster herpes virus (OsHV-1) infection (17). Their expression variation maybe evolutionarily critical for the functional divergence of Family I84 genes.

Family I84 genes expression increased in response to pathogenic bacteria and parasite; *Vibrio* challenge led to the elevation of expression for 8 of 14 Family I84 genes in the resistance group compared to the sensitive group. CvSI-1 was the only gene that was highly upregulated (day 15 post challenge) in hemocytes of adult oysters challenged with the pathogen of larval shellfish-*V. tubiashii*, even if it does not cause mortality in adult oysters (14). Meanwhile, expression of *cvSI-1* and *cvSI-2* were upregulated by injection of *R. crassostreae*, the bacterial pathogen of the Roseovarius oyster disease (ROD) in eastern oysters (41). In addition, the expression patterns of the Family I84 genes are inducible by salinity stresses and pathogen-associated molecular pattern stimulations (9, 11). These observation give emphasis to their involvement in function in host defense of related species.

The specific expression of Family I84 genes in the digestive gland should be strictly regulated due to they are hardly expressed in gills. Although no motifs were shared among promoter regions of Family I84 members, it was found that the cis-regulatory motifs of duplicated genes tend to diversify in yeast and mussel (36, 42). On the contrary, several transcription factor binding sites were shared among Family I84 genes, most of which are in response to physiological and pathological stimuli. In the future, functional identification of transcription factors is crucial for the regulation of target gene expression for their diversified function and evolution.

In conclusion, the full repertoire of Family I84 genes shared same gene structure, mature peptide of these member compromise 12 conserved cysteine residues with codon frequency preservation of 5 cysteine. Loss of a predicted signal peptide for scSI-7 and a premature termination for the scSI-14 increased their molecular diversity and may drive their silence compared to previous Family I84 genes. Remarkably, most razor clam Family I84 genes should experience lineage specific expansion by tandem duplication. Differential expression arise for tandem duplicated gene pairs, while high correlation of gene expression observed between non-tandem duplicated genes. These results provides a comprehensive understanding for the evolution of Family I84 protease inhibitor and their function in host defense.

## Data Availability Statement

The original contributions presented in the study are included in the article/**Supplementary Material**. Further inquiries can be directed to the corresponding author.

## Author Contributions

QX and ZL conceived and designed the study. SL performed gene identification and the statistical analyses. YL conducted transcriptome assembly and gene expression analysis. JL and JM participate in sequence verification. SL and QX wrote and revised the manuscript. All authors contributed to the article and approved the submitted version.

## Funding

This work was financially supported by National Natural Science Foundation of China (32002428, 32073010), “3315” Innovative Team of Ningbo City, the Fundamental Research Funds for Zhejiang Province Universities and China Agriculture Research System of MOF and MARA.

## Conflict of Interest

The authors declare that the research was conducted in the absence of any commercial or financial relationships that could be construed as a potential conflict of interest.

## Publisher’s Note

All claims expressed in this article are solely those of the authors and do not necessarily represent those of their affiliated organizations, or those of the publisher, the editors and the reviewers. Any product that may be evaluated in this article, or claim that may be made by its manufacturer, is not guaranteed or endorsed by the publisher.
