# Supplementary figures and images for: Genome Wide Identification and Expression Profiling Indicate Expansion of Family I84 Protease Inhibitor via Gene Tandem Duplication and Divergence in Razor Clam Sinonovacula constricta
Source: Front Immunol. 2022 Jun 1;13:907274. doi: 10.3389/fimmu.2022.907274 (PMC9198434; doi:10.3389/fimmu.2022.907274)

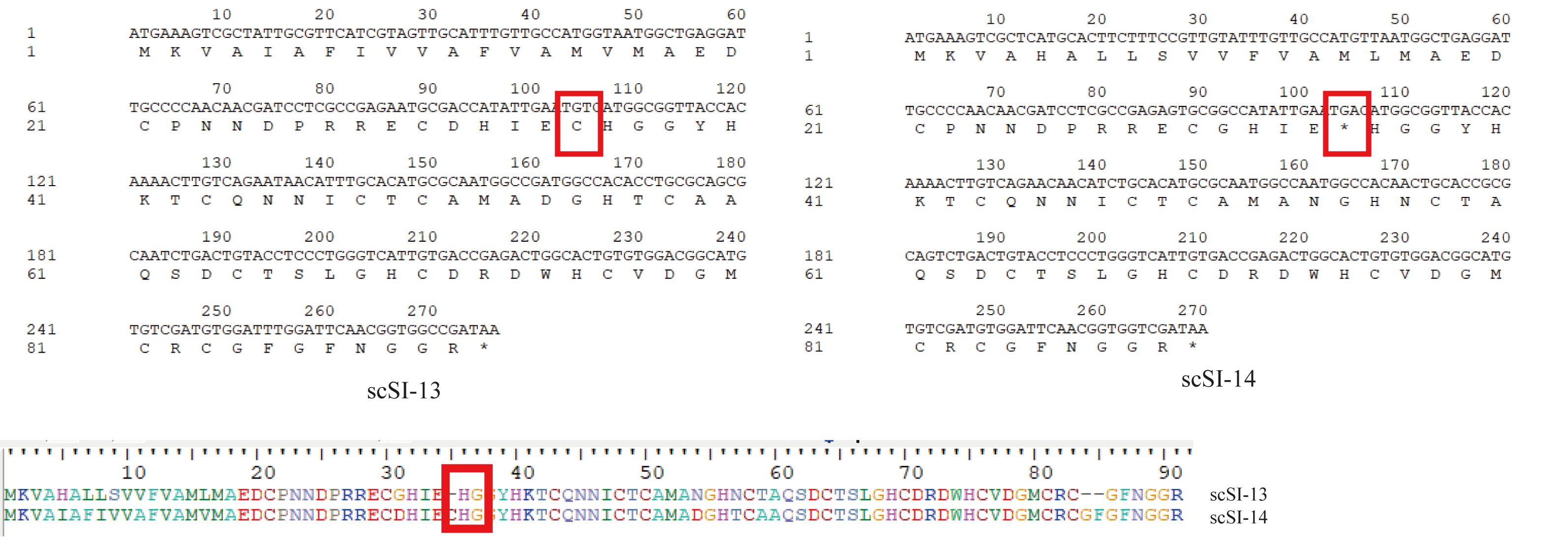

Supplement: Supplementary file 1 [file Image_1.tif]

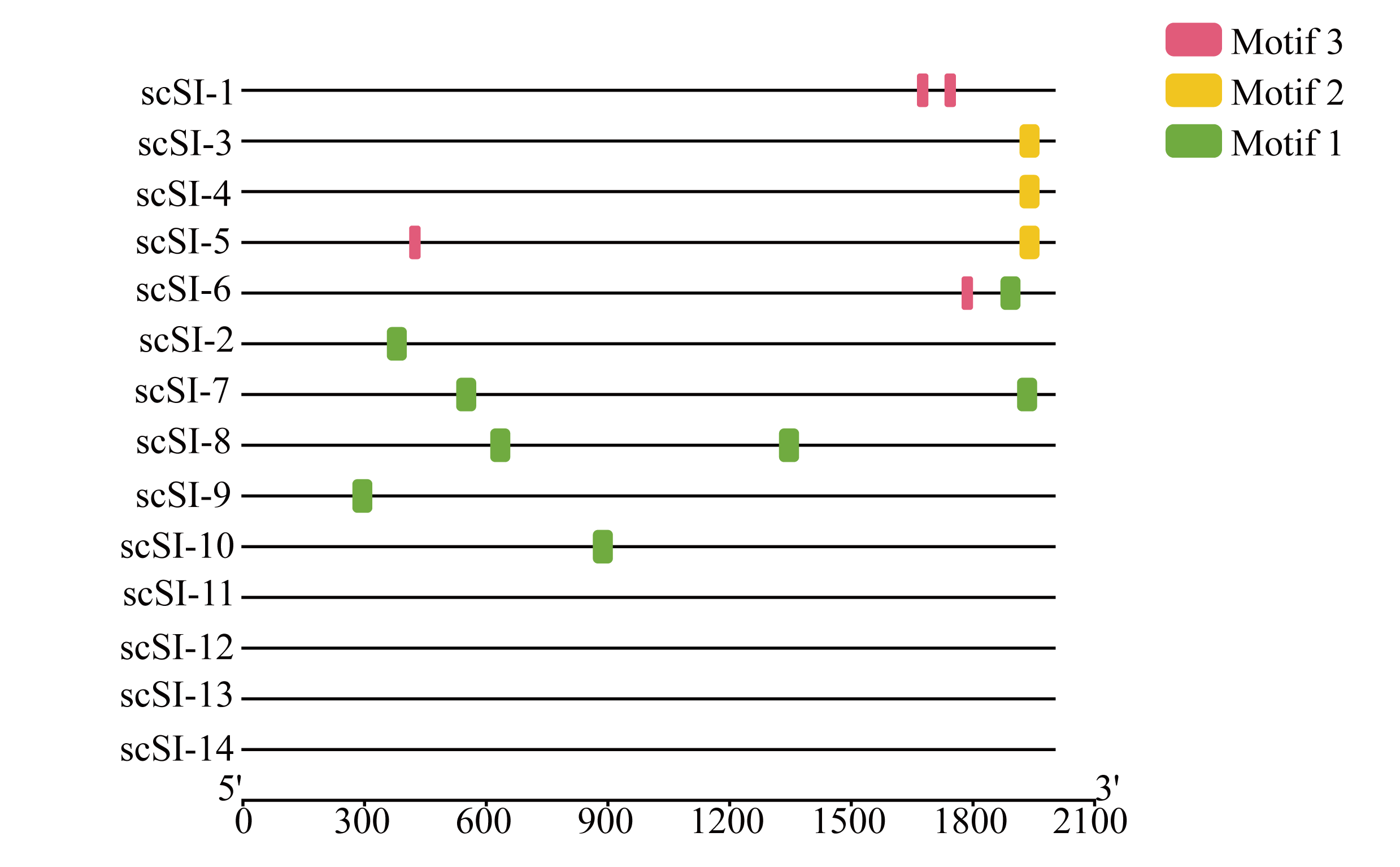

Supplement: Supplementary file 2 [file Image_2.tif]
