# Supplementary material for: Genome Wide Identification and Expression Profiling Indicate Expansion of Family I84 Protease Inhibitor via Gene Tandem Duplication and Divergence in Razor Clam Sinonovacula constricta
Source: Front Immunol. 2022 Jun 1;13:907274. doi: 10.3389/fimmu.2022.907274 (PMC9198434; doi:10.3389/fimmu.2022.907274)
Supplement: Supplementary file 6 [file Table_4.docx]

Supplementary table 4 Statistics of Transcription factor binding sites and transcription factors

|  | TFBS | TF |
| --- | --- | --- |
| scSI1 | 20 | 15 |
| scSI-3 | 41 | 29 |
| scSI-4 | 31 | 17 |
| scSI-5 | 44 | 28 |
| scSI-6 | 29 | 16 |
| scSI-2 | 28 | 14 |
| scSI-7 | 28 | 16 |
| scSI-8 | 24 | 16 |
| scSI-9 | 33 | 18 |
| scSI-10 | 53 | 28 |
| scSI-11 | 31 | 20 |
| scSI-12 | 31 | 20 |
| scSI-13 | 27 | 15 |
| scSI-14 | 47 | 14 |
